# Novel approach to orbital hard- and soft-tissue symmetry analysis: a pilot validation

**DOI:** 10.1186/s13005-025-00538-1

**Published:** 2025-10-09

**Authors:** Ilya Tsiklin, Joeri Meyns, Robin Willaert, Thanatchaporn Jindanil, Nermin Morgan, Flavia Preda, Eman Shaheen, Reinhilde Jacobs

**Affiliations:** 1 OMFS IMPATH Research Group, Department of Imaging and Pathology, Faculty of Medicine, University of Leuven, Kapucijnenvoer 33, Leuven, 3000 Belgium; 2 Department of Oral and Maxillofacial Surgery, University Hospitals Leuven, Leuven, Belgium; 3 Department of Oral and Maxillofacial Surgery, General Hospital St-Jan Genk, Genk, Belgium; 4 Department of Radiology, Faculty of Dentistry, Chulalongkorn University, Bangkok, Thailand; 5 Department of Dental Medicine (DENTMED), Karolinska Institutet, Stockholm, Sweden

**Keywords:** Orbital symmetry, Hard- and soft-tissue structures, Model mirroring and registration, Surface-based symmetry analysis

## Abstract

**Background:**

Volume-based orbital symmetry analysis can hardly explain a particular orbital structure’s involvement and contribution to asymmetry. This study aimed to validate a novel approach to orbital surface-based hard- and soft-tissue symmetry analysis, providing comprehensible and clinically applicable interpretation. CT-based segmentation of the orbital hard- and soft-tissue structures was followed by model mirroring, global registration, and surface-based symmetry analysis, with both qualitative and quantitative interpretations in twenty young, healthy subjects.

**Results:**

The entire soft-tissue model’s global symmetry was higher compared to the entire hard-tissue volume (*p* = 0.027), reflecting the potential of the soft tissue to compensate for bone-structure asymmetry. No significant difference in symmetry parameters was found for particular orbital walls (*p* = 0.803) and intra-orbital soft-tissue structures (*p* = 0.256). The strong negative correlation between relative global and local symmetry parameters and absolute root mean square (RMS) distance values demonstrates the reliability of the suggested novel approach. Correlations between orbital walls symmetry parameters were higher compared to soft-tissue orbital structures.

**Conclusion:**

Aside from the accurate RMS distance calculation, the global and local symmetry analysis presented in this pilot study offers valuable, reliable, and comprehensible information on the contribution of particular orbital hard- and soft-tissue structures to facial symmetry.

## Background

Minor facial asymmetry is generally considered natural and acceptable, and a correlation between hard- and soft-tissue morphology has been previously reported. Asymmetry of the lower face is observed more often than in the upper and midface areas; however, periorbital soft tissues can be more sensitive to minimal changes and side-to-side differences [1–4].

CT-based 3D rendering, segmentation, and mirroring techniques have been widely used for orbital imaging and virtual surgical planning in patients with orbital trauma or thyroid-associated ophthalmopathy [5–11]. Multiple studies presented orbital symmetry evaluation based on orbital volume changes; however, volume-based orbital symmetry analysis can hardly explain a particular orbital structure’s involvement and contribution to asymmetry [12–14].

Surface-based symmetry analysis, which measures the deviation between registered surfaces, has been successfully applied for qualitative and quantitative facial hard- and soft-tissue symmetry assessment. This method has demonstrated high accuracy and reproducibility, providing a reliable basis for our research [1–4, 17–19]. Hypothetically, detailed surface-based analysis of the various orbital hard- and soft-tissue surfaces can provide valuable information on the differential involvement and contribution of particular anatomical structures to orbital asymmetry.

This pilot study aims to validate a novel approach to orbital surface-based hard- and soft-tissue symmetry analysis. The primary goal is to provide a comprehensible, qualitative, and quantitative interpretation that can be potentially applied in clinical settings, thereby enhancing the practicality of the research.

## Methods

### Study design

Computed tomography (CT) scans of twenty young, healthy subjects (11 male; 9 female; mean age 15.8 ± 1.05 years) previously obtained for diagnostic purposes and orthodontic treatment planning were selected for this study. Any history of orbital trauma, pathology, or congenital deformity was considered an exclusion criterion.

### Image acquisition

Twenty non-contrast CT-scans, including both orbits and midface bone structures were obtained (slice thickness – 0.6 mm, pixel spacing – 0.4 × 0.4 mm, Siemens, Somatom Force, Germany).

### Image segmentation

**Fig. 1:**
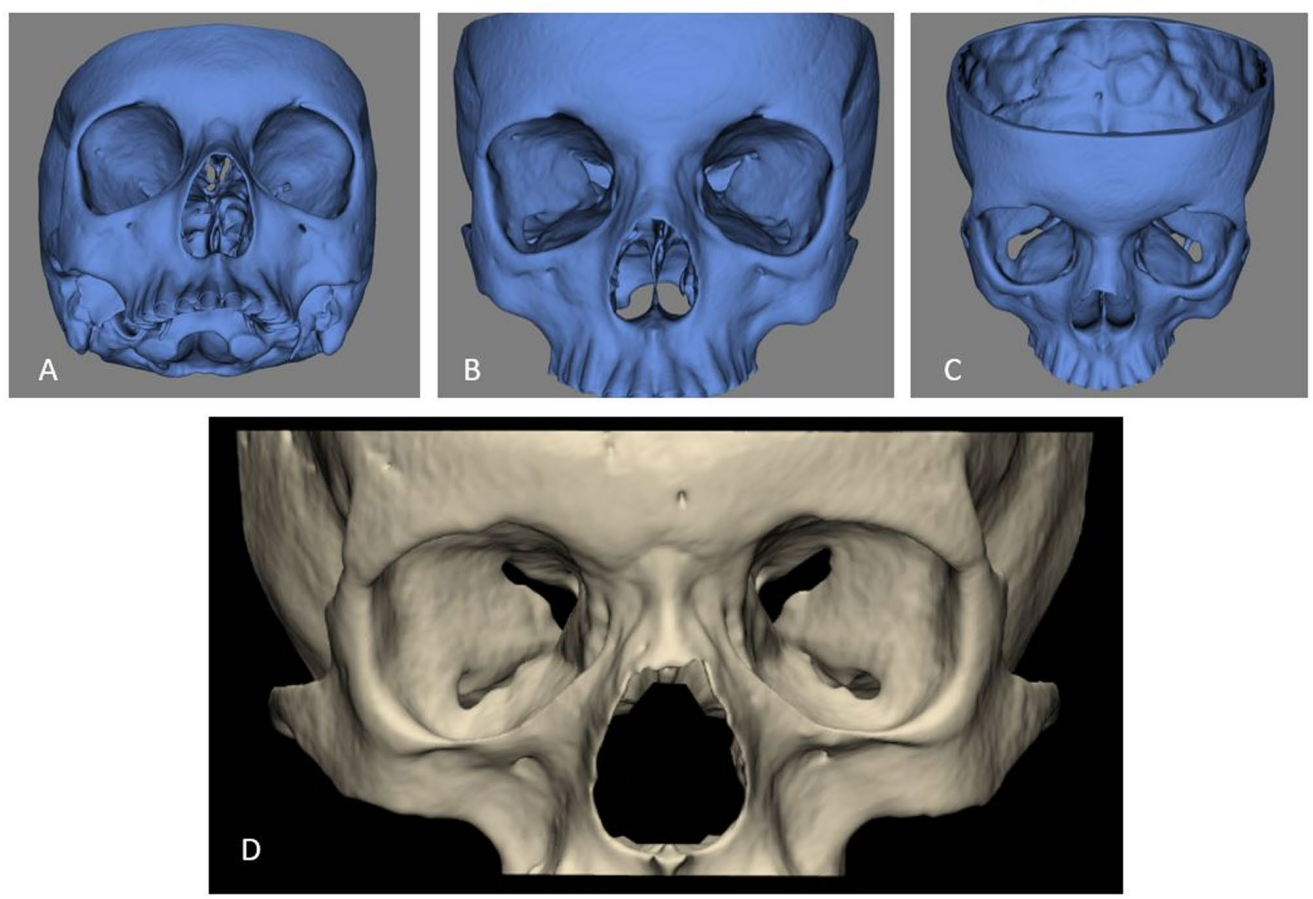
(**A - C**) Automated AI-based skull segmentation (“Virtual Patient Creator”, Relu BV, Leuven, Belgium) (**D**) Final cut and refinement of the orbital hard-tissue model (3-Matic Software, version 18, Materialise N.V., Leuven, Belgium)

The automated segmentation of the skull was performed using the previously validated CNN-based online cloud tool Virtual Patient Creator (ReLu BV, Leuven, Belgium) [23]. The AI model was initially trained and developed for the maxillofacial complex, including the entire skull, orbit, and maxilla, using both CT and CBCT data. Further training and validation were conducted with additional 144 CBCT scans. The training results demonstrated a high DSC value of 92.6% and an IoU of 0.86, indicating a strong accuracy in automated bone segmentation [21–23]. The segmentation quality was assessed in Mimics Innovation Suite Version 24 (Materialise N.V., Leuven, Belgium). Created three-dimensional (3D) orbital hard-tissue model in Standard Tessellation Language (STL) format was imported into 3-Matic Software (version 18, Materialise N.V.,Leuven, Belgium) for the final hard-tissue model cut (alveolar process of the maxilla and nasal septum were removed), refinement, registration, and further qualitative and quantitative analysis. (Fig. 1).

**Fig. 2:**
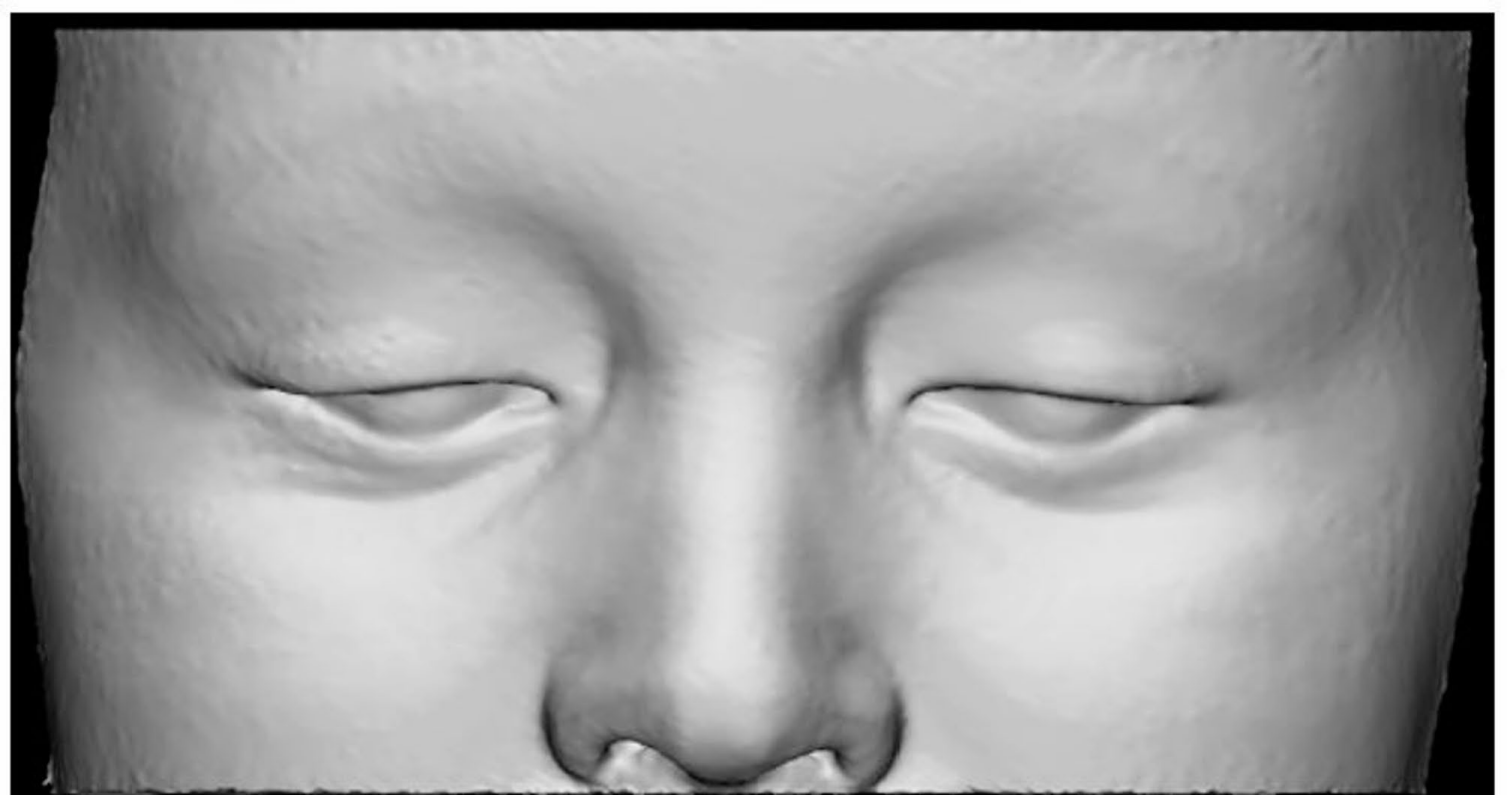
Automated threshold-based peri-orbital soft-tissue model segmentation (Mimics Innovation Suite (version 24, Materialise N.V.,Leuven, Belgium)

Periorbital soft-tissue model was automatically segmented using Mimics Innovation Suite Version 24 (Materialise N.V., Leuven, Belgium) using threshold-based approach (-700; 225 HU). Automated segmentation of the soft-tissue complex was followed by the final cut and refinement of the model in 3-Matic Software, version 18 (Materialise N.V., Leuven, Belgium) (Fig. 2).

**Fig. 3:**
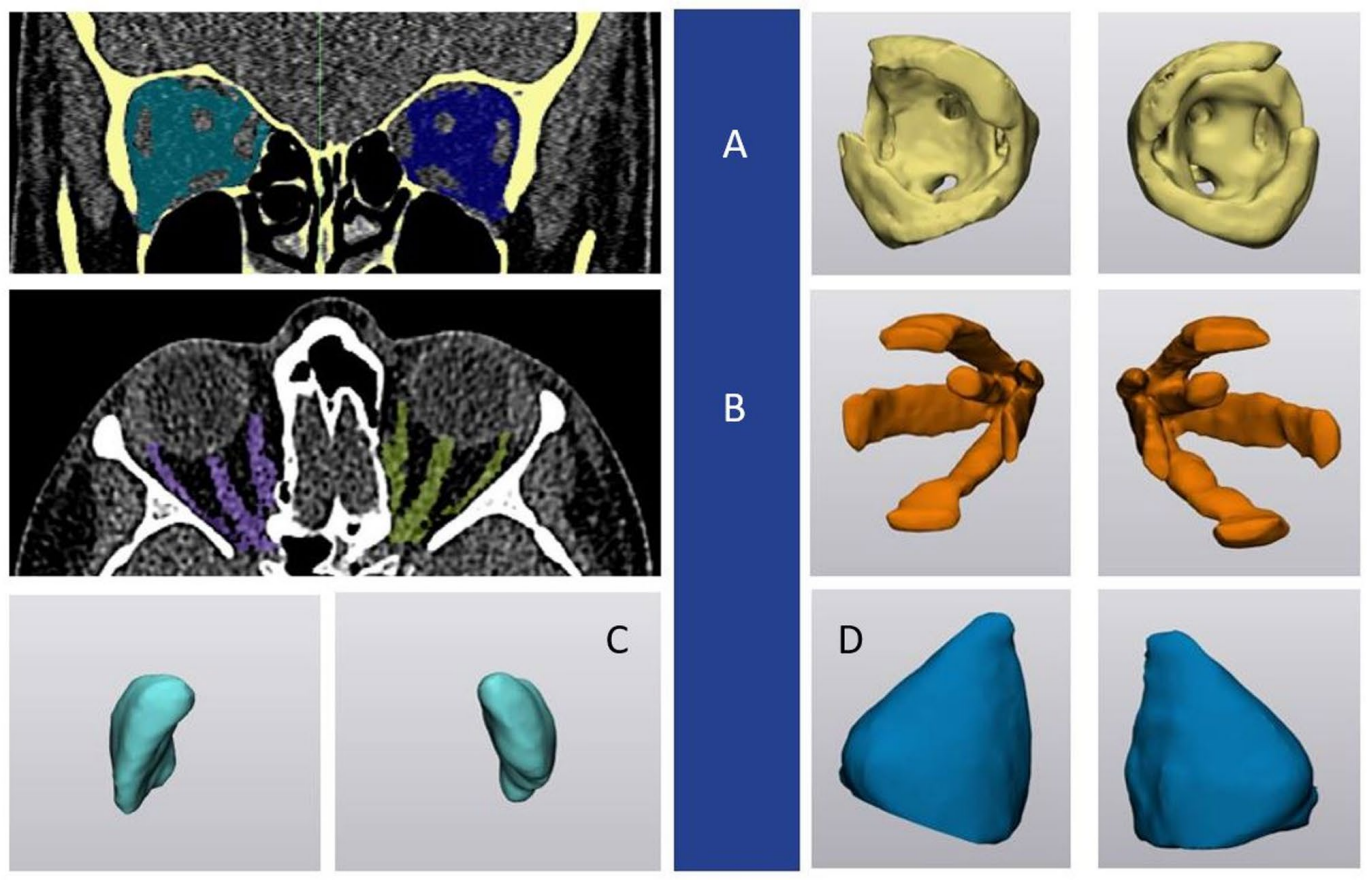
Semi-automated intra-orbital soft-tissue structures segmentation: orbital fat (**A**), extraocular muscles (**B**), lacrimal glands, total orbital volume (**C**,** D**) (Mimics Innovation Suite; version 24, Materialise N.V.,Leuven, Belgium)

Segmentation of the intra-orbital soft-tissue structures, including orbital fat (OF), extraocular muscles (EOM), optic nerve, lacrimal glands (LG), and total orbital volume (TOV) was performed using a semi-automated threshold-based segmentation using previously reported thresholding parameters [2, 27] (Mimics Innovation Suite, version 24, Materialise N.V.,Leuven, Belgium). Orbital septum was chosen as the anatomic boundary for intra-orbital (retroseptal) OF segmentation (Fig. 3).

Orbital soft-tissue structures segmentation methods are summarized in Table 1.

**Table 1:** Orbital Soft-Tissue structures segmentation methods and thresholding parameters

Anatomical Structure	Orbital Fat	Extraocular Muscles + Optic Nerve	Lacrimal Gland	Total Orbital Volume	Periorbital Soft Tissues
**Segmentation Methods**	Dynamic region-growth tool + Manual delineation	Multislice threshold-based segmentation + Manual delineation	Multislice threshold-based segmentation + Manual delineation	Multislice threshold-based segmentation + Manual delineation	Automated threshold-based segmentation
**CT Thresholding Parameters**	[-200; 15 HU]	[-30; 205 HU]	[-700; 200 HU]	[-700; 225 HU]	[-700; 225 HU]

*HU: Hounsfield Units

The volume measurements of all segmented paired soft-tissue structures were performed and right-to-left comparisons using two-sample t-test were applied.

### Anatomical mapping of the hard- and soft-tissue models

**Fig. 4:**
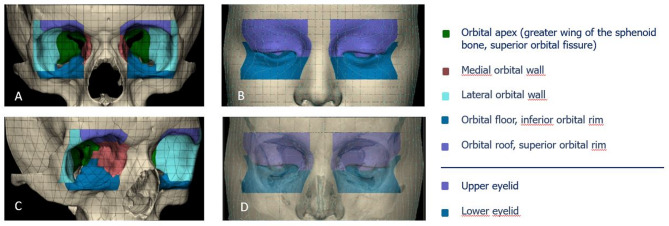
Orbital hard-tissue (**A**,** C**) and periorbital soft-tissue (**B**,** D**) model anatomical mapping (3-Matic Software, version 18, Materialise N.V., Leuven, Belgium)

We used a grid surface segmentation tool (3-Matic Software, version 18, Materialise N.V., Leuven, Belgium). The surface of the 3D object was automatically divided into equal grid cells with a surface area of 5 × 5 mm for further global and local symmetry analysis. The total area surface of each 3D model was automatically calculated. Anatomical mapping of the hard-tissue model included orbit differentiation into five colored anatomical zones representing orbital apex (greater wing of the sphenoid bone and superior orbital fissure), medial orbital wall, lateral orbital wall, orbital floor and inferior orbital rim, orbital roof and superior orbital rim. The periorbital soft-tissue anatomical map included upper and lower eyelid zones overlapping the orbital rims. Surface area of each anatomical zone was calculated by selecting the corresponding grid cells and right-to-left comparison of the area surface using two-sample t-test was performed (Fig. 4).

### Image mirroring and model registration

**Fig. 5:**
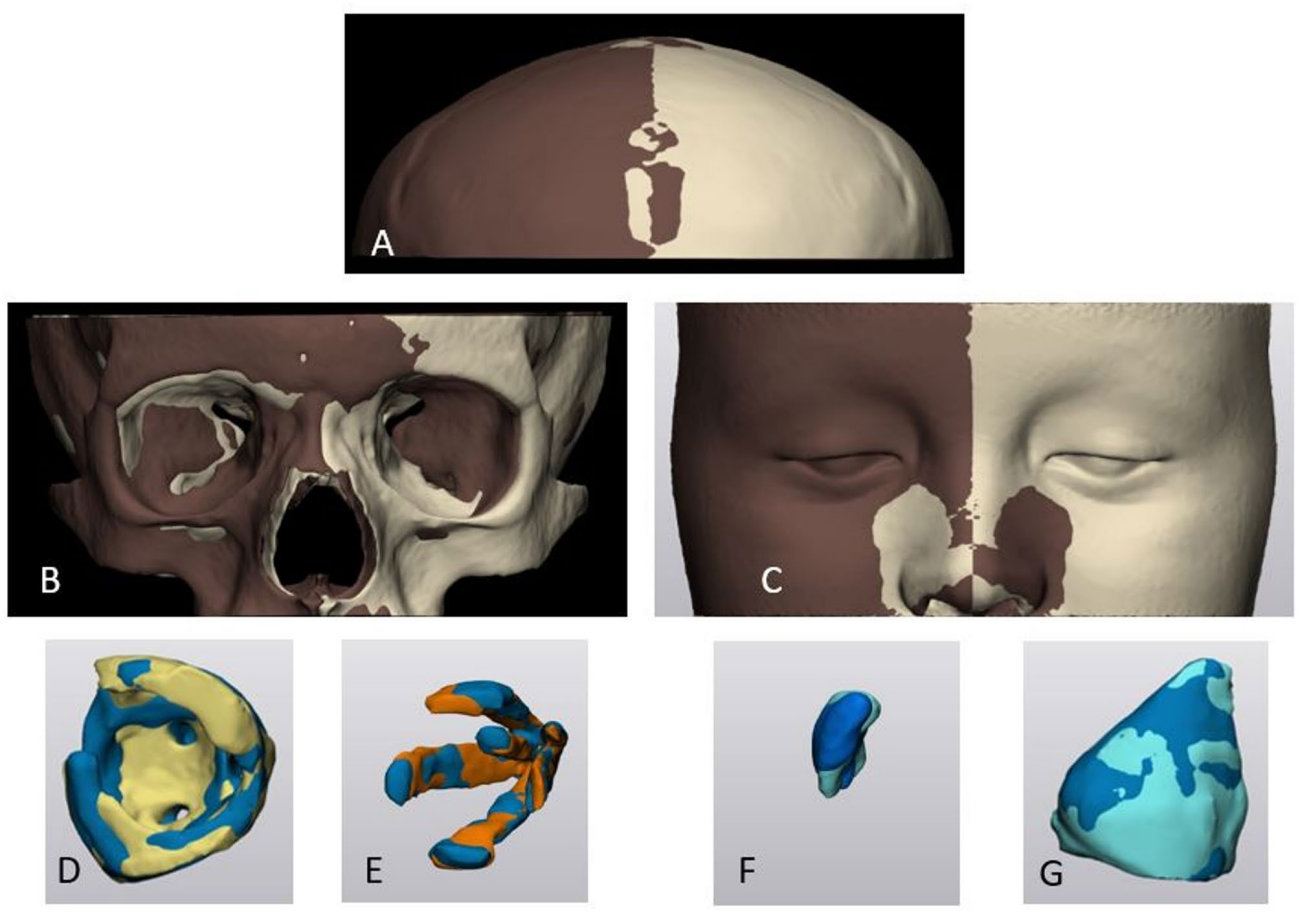
Orbital hard- and soft-tissue structures mirroring and global registration: (**A**) reference skull vault model; orbital hard-tissue (**B**) and periorbital soft-tissue (**C**) model registration, orbital fat (**D**), extraocular muscles (**E**), lacrimal gland (**F**), total orbital volume (**G**) registration (3-Matic Software, version 18, Materialise N.V.,Leuven, Belgium)

Hard- and soft-tissue models were mirrored along automatically determined midsagittal plane followed by automated iterative surface-based global registration of the true and mirrored model until reaching the least point-to-point distance (mm) between the two model surfaces. With a purpose to enhance accuracy of the model mirroring and registration the iteration-based global registration of the true and mirrored hard- and soft-tissue models was accomplished along the reference hard-tissue model – upper part of the skull vault (3-Matic Software, version 18, Materialise N.V., Leuven, Belgium). Figure 5.

### Orbital hard- and soft-tissue symmetry analysis

**Fig. 6:**
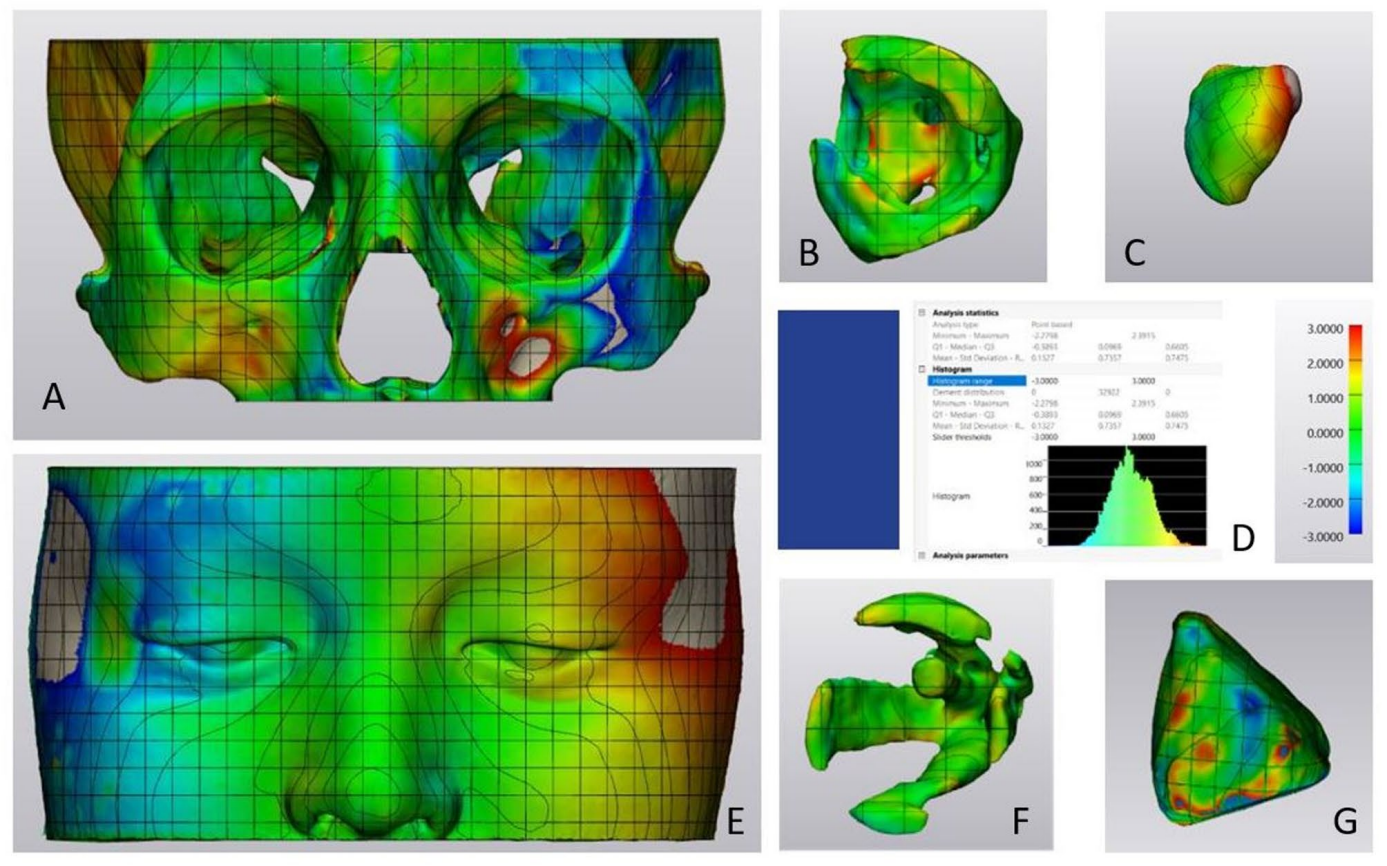
Orbital hard- and soft-tissue symmetry analysis: color-coded map calculation - orbital hard-tissue (**A**), orbital fat (**B**), lacrimal glands (**C**), deviation histogram (**D**), periorbital soft-tissue (**E**), extraocular muscles (**F**), total orbital volume (**G**) (3-Matic Software, version 18, Materialise N.V., Leuven, Belgium)

Morphological symmetry of the registered true and mirrored models was analyzed using part comparison analysis tool (3-Matic Software, version 18, Materialise N.V.,Leuven, Belgium). Color-coded map of the model surface graphically represented a histogram of positive and negative deviation values between the two surfaces (mean point-to-point distance, standard deviation, and Root-Mean-Square (RMS) were automatically calculated by the software). According to the previously reported results on facial hard- and soft-tissue symmetry analysis and susceptibility of the periorbital soft-tissue structures to minor asymmetry and side-to-side differences the deviation [1–3], deviation values with the range ± 1 mm were considered clinically non-significant for both orbital hard- and soft-tissue structures (green color code) (3-Matic Software, version 18, Materialise N.V.,Leuven, Belgium) (Fig. 6).

Created color-coded maps were automatically segmented into clearly demarcated zones based on the same thresholding settings for further global and local symmetry analysis.

**Fig. 7:**
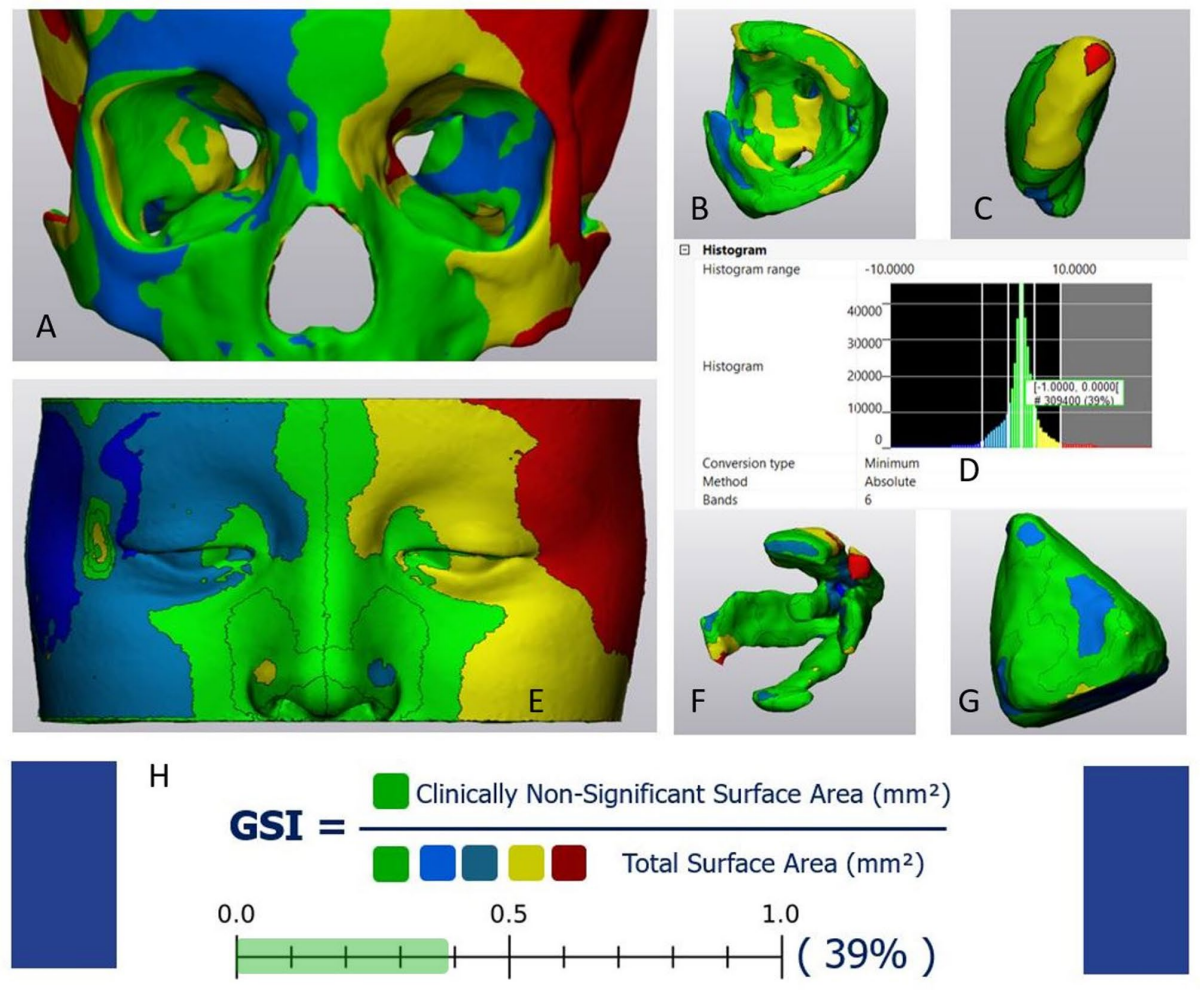
Orbital hard- and soft-tissue global symmetry analysis: color-coded map segmentation and *Global Symmetry Index* calculation: orbital hard-tissue (**A**), orbital fat (**B**), lacrimal gland (**C**), deviation distance histogram (**D**), periorbital soft-tissue (**E**), extraocular muscles (**F**), total orbital volume (**G**), Global Symmetry Index calculation (3-Matic Software, version 18, Materialise N.V.,Leuven, Belgium)

*Global Symmetry Index (GSI)* of the hard- and soft orbital tissue structures was calculated as the ratio between the surface area with clinically non-significant deviations (mm^2^) and the total surface area of the 3D model. Global symmetry of the hard-tissue model and the entire soft-tissue model was analyzed in all twenty subjects, while particular orbital walls and intra-orbital soft-tissue structure’s symmetry was evaluated in ten subjects (5 male; 5 female) (Fig. 7).

**Fig. 8:**
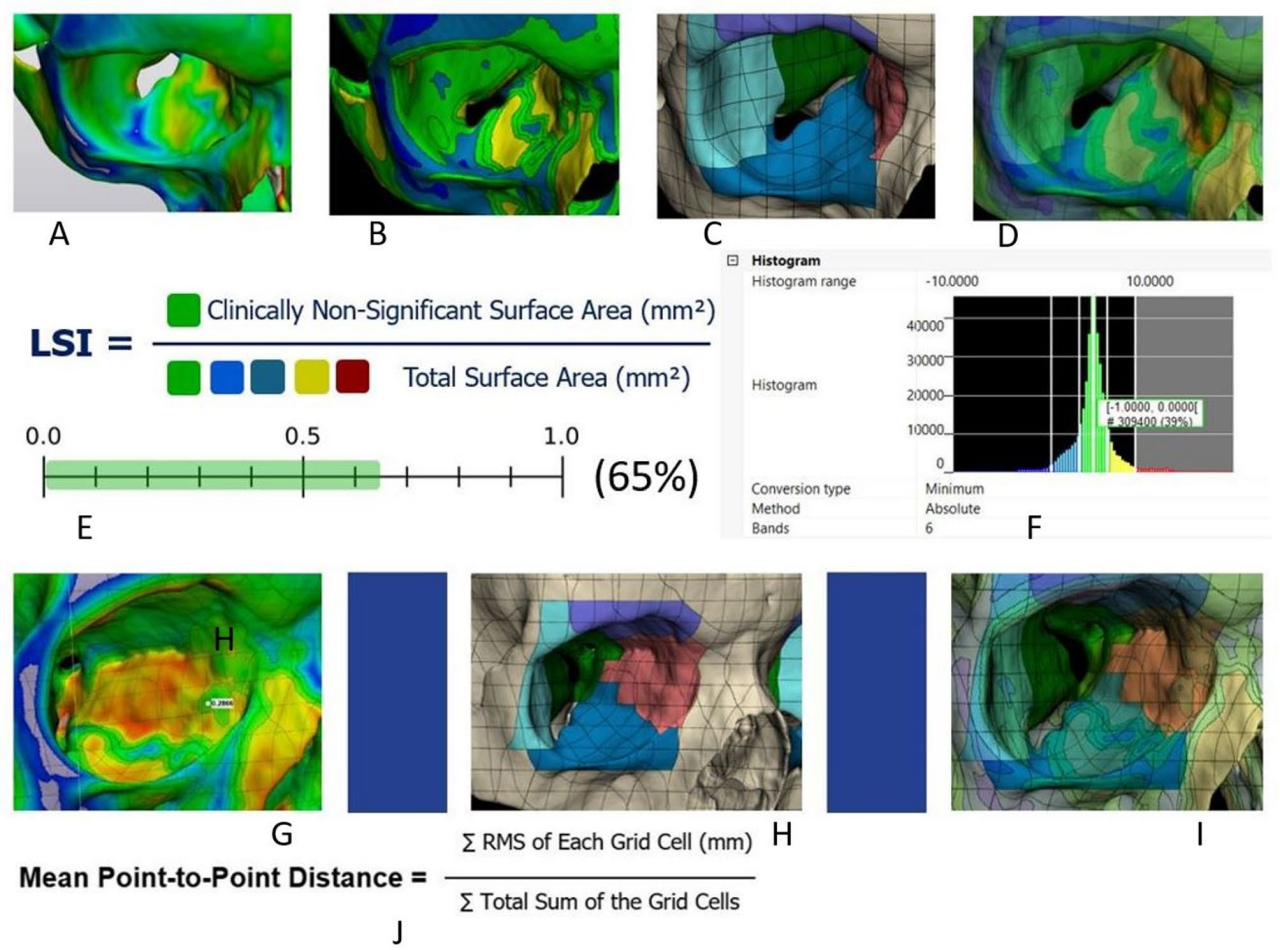
Orbital hard-tissue local symmetry analysis and *Local Symmetry Index* calculation: orbital floor deviation map (**A**), orbital floor deviation map segmentation (**B**), orbital hard-tissue anatomical map (**C**,** H**), superimposition of the hard-tissue anatomical map and semitransparent segmented deviation map (**D**,** I**), Local Symmetry Index calculation (**E**), deviation distance histogram (**F**), medial orbital wall local deviation distance calculation (**G**), local mean point-to-point distance calculation (**J**) (3-Matic Software, version 18, Materialise N.V.,Leuven, Belgium)

Local symmetry analysis was performed for the same group of ten observations (5 male; 5 female) using segmented color-coded maps with the grid cell segmentation. *The local Symmetry Index (LSI)* was calculated as the ratio between the surface area of the particular orbital wall or periorbital soft-tissue surface with clinically non-significant deviations (mm^2^) and the total surface area of the particular surface (mm^2^). Local symmetry analysis also included calculating the RMS point-to-point distance for the particular anatomical zone. It was calculated as the ratio between the sum of the RMS values of the grid cells referring to the particular anatomical zone and the total surface area of this zone (Fig. 8).

### Statistical analysis

Statistical analysis was performed using RStudio software package (version 4.3.1; RStudio, Boston, MA, USA). Prior to conducting this pilot study the a-priori sample size calculation was performed. The sample size of size of 20 produced a power of 80%, assuming an anticipated effect size of 0.5 and significance level of 5% (α = 0.05).The descriptive statistics included minimum, maximum, mean, and standard deviation (SD). Shapiro–Wilk test was used to check for normality of the data. The mean deviation differences for all morphologic regions were analyzed by means of two-sample t-test, one-way analysis of variance (ANOVA) with multiple comparisons (Tukey test) or non-parametric alternative methods (Wilcoxon test; Kruskal-Wallis test). To assess the intra-observer error the entire process including semi-automated segmentation of the soft orbital tissue structures, manual refinement, mirroring and registration procedure, calculation of mean and RMS point-to-point distance between the two model surfaces, was repeatedly performed by the same operator two weeks after the first procedure. Pearson correlation coefficient was calculated to assess the association between symmetry of the various hard- and soft-tissue orbital structures. The level of significance was defined as α = 0.05.

## Results

The quality and readability of the CT scans were considered appropriate for further segmentation and analysis. Automatically segmented hard- and soft-tissue models required minimal refinements. Volumetric parameters of the paired orbital soft-tissue structures are shown in Table 2.

**Table 2:** Volumetric characteristics of the segmented orbital soft-tissue structures

Segmented Orbital Structure	Volume Right Side (cm^3^) Mean Volume ± SD	Volume Left Side (cm^3^) Mean Volume ± SD	*p*-value*
Orbital Fat	11.8 ± 1.5	11.9 ± 1.9	0.72
Extraocular Muscles + Optic Nerve	4.23 ± 0.9	4.29 ± 1.1	0.74
Lacrimal Gland	0.73 ± 0.2	0.71 ± 1.8	0.61
Total Orbital Volume	22.3 ± 1.1	22.5 ± 1.4	0.62

*Level of significance *p* < 0.05

Right-to-left comparison of the anatomical maps of the orbital walls and periorbital soft-tissue surface demonstrated no significant difference Table 3.

**Table 3:** Right-to-Left area surface comparison of the orbital Hard- and Soft-Tissue structures

Anatomical Zone	*p*-value (Two-sample t-test)*
Orbital Apex (greater wing of the sphenoid bone, superior orbital fissure)	*p* = 0.74
Medial Orbital Wall	*P* = 0.85
Lateral Orbital Wall	*P* = 0.62
Orbital Floor, Inferior Orbital Rim	*p* = 0.75
Orbital Roof, Superior Orbital Rim	*p* = 0.56
Upper Eyelid	*p* = 0.75
Lower Eyelid	*p* = 0.67

*Level of significance *p* < 0.05

Least point-to-point distances reached during the global registration of the true and mirrored hard- and soft-tissue models are presented in Table 4.

**Table 4:** Point-to-Point distance between true and mirrored Hard- and Soft-Tissue models

Orbital Structure	Mean Point-to-Point Distance (mm)	Standard Deviation
Orbital Hard-Tissue Model	0.37	0.09
Periorbital Soft-Tissue Model	0.15	0.14
Orbital Fat	0.17	0.55
Extraocular Muscles	0.21	0.7
Lacrimal Gland	0.11	0.09
Total Orbital Volume	0.19	0.11

**Fig. 9:**
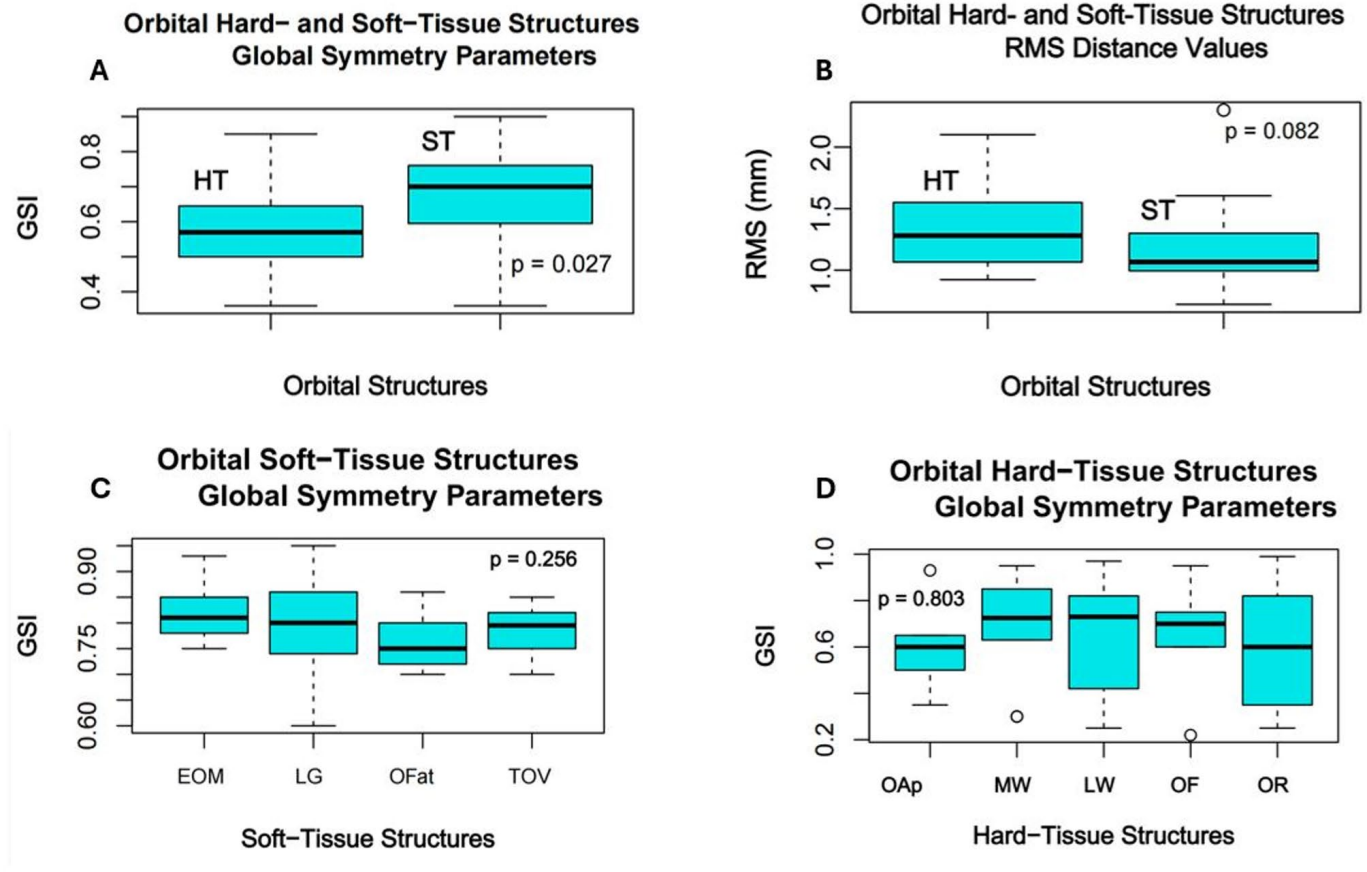
Orbital hard- and soft-tissue symmetry analysis: orbital hard- and soft-tissues GSI comparative analysis (**A**), orbital hard- and soft-tissues RMS comparative analysis (**B**); orbital soft-tissue structures GSI comparative analysis (**C**); orbital hard-tissue structures symmetry comparative analysis (**D**). *Level of significance *p* < 0.05

The hard-tissue orbital model demonstrated significantly lower global symmetry than the periorbital soft-tissue model (*p* = 0.028), while the difference in the hard- and soft-tissue models’ RMS values was insignificant (*p* = 0.082). According to the results of the one-way ANOVA test there was no significant difference between the symmetry parameters of particular intra-orbital soft-tissue structures (*p* = 0.256) and certain orbital surfaces (*p* = 0.803) (Fig. 9).

**Fig. 10:**
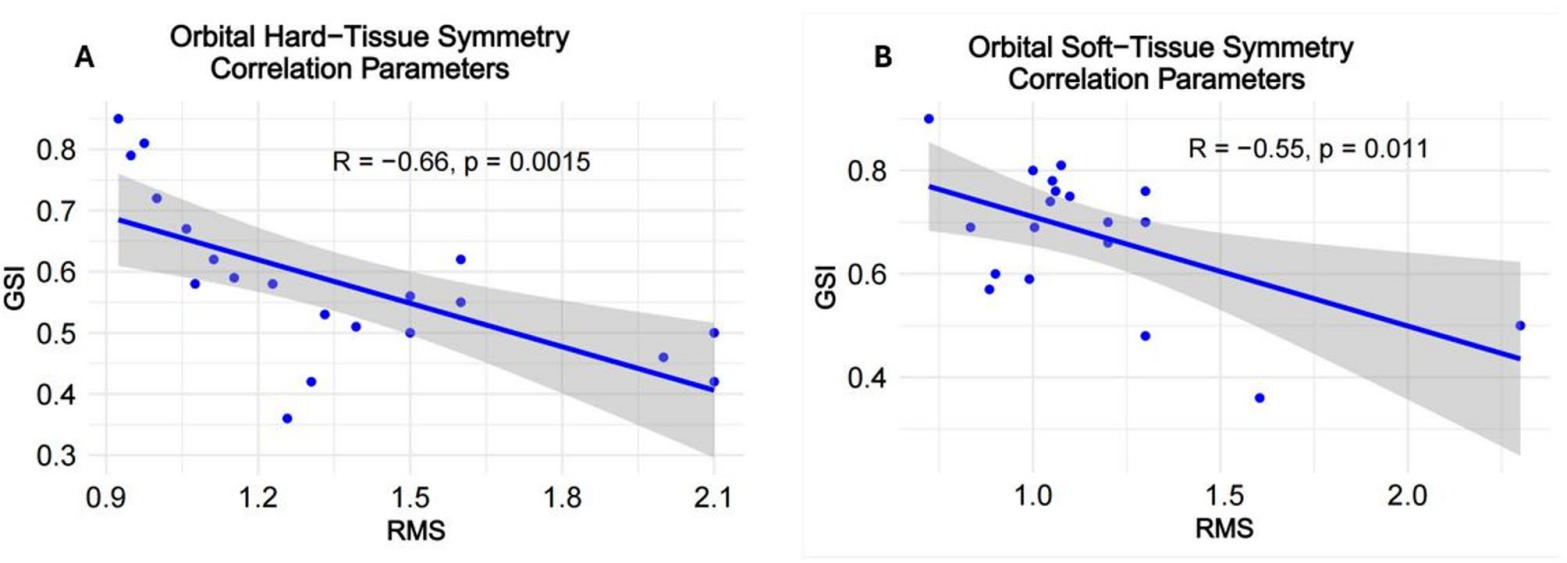
Pearson correlation coefficient values for RMS and Global Symmetry assessment parameters for the orbital hard-tissue (**A**) and soft-tissue (**B**) structures

Pearson correlation coefficient values for RMS and Global Symmetry assessment parameters for orbital hard- and soft-tissue structures are shown in the correlation plots below (Fig. 10).

**Fig. 11:**
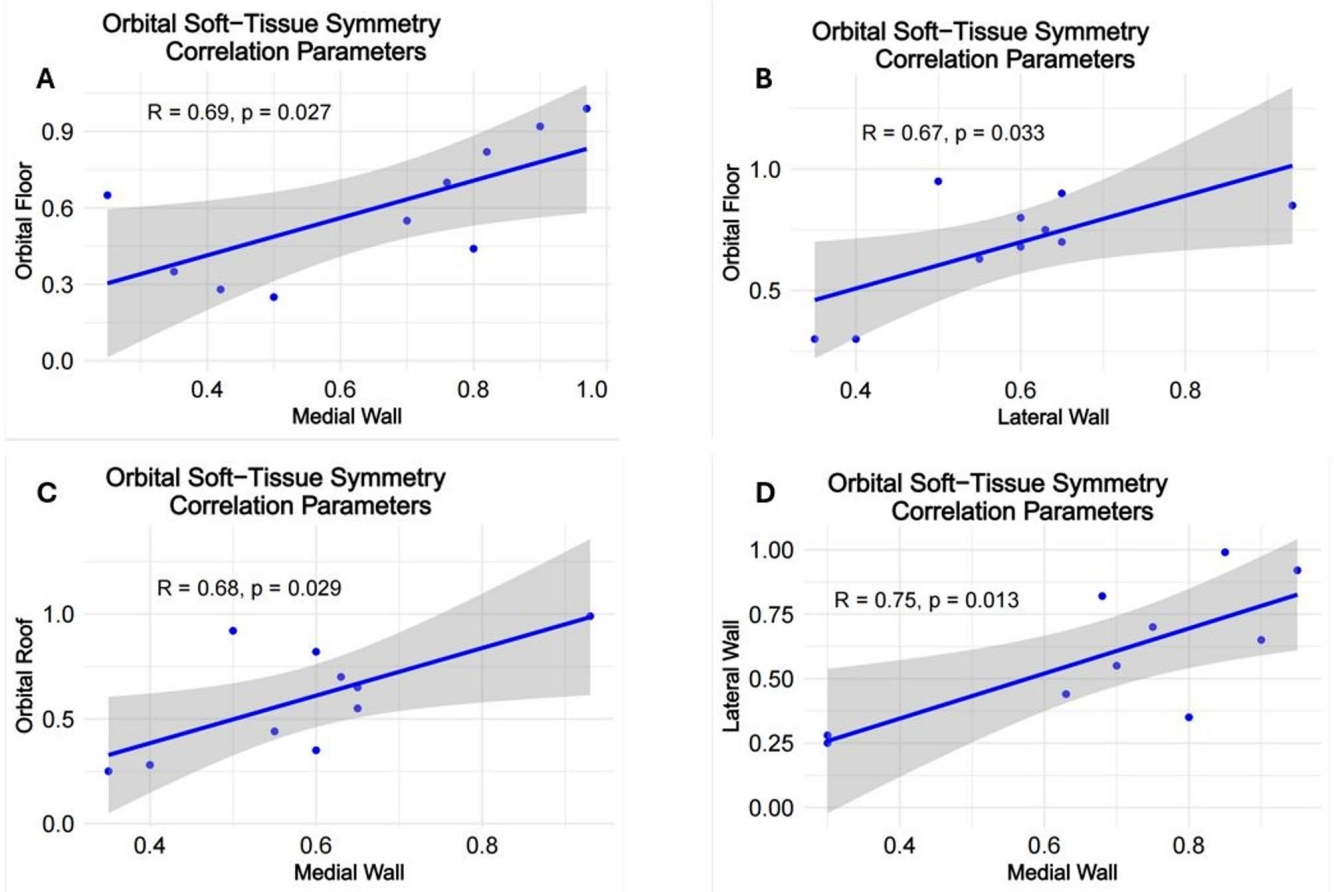
Correlations between symmetry parameters of the orbital walls (**A**) Orbital Floor-Medial Wall; (**B**) Orbital Floor – Lateral Wall; (**C**) Orbital Roof – Medial Wall; (**D**) Lateral Wall – Medial Wall

**Fig. 12:**
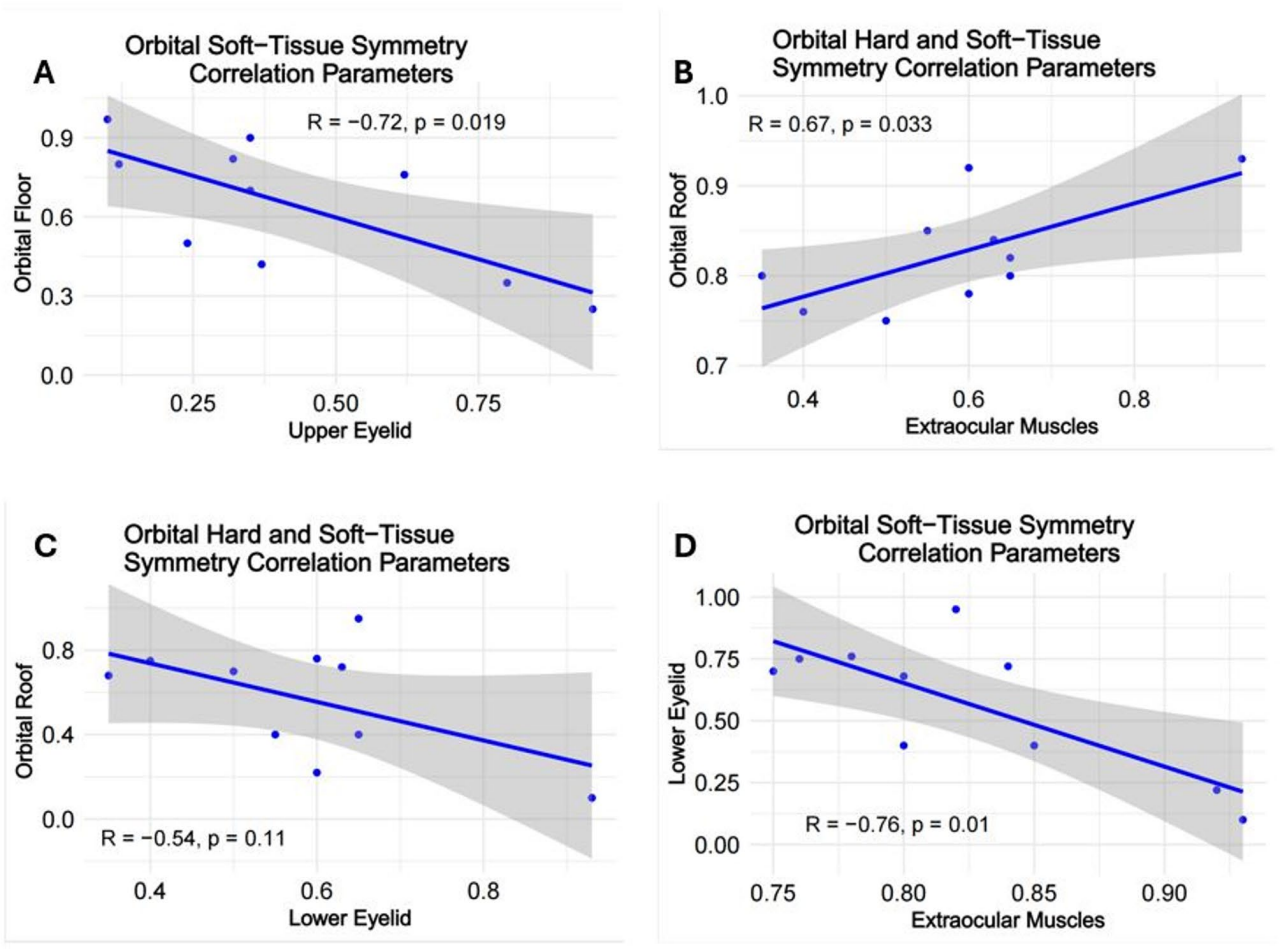
Correlations between symmetry parameters of the orbital hard- and soft-tissue structures: (**A**) Orbital Floor-Upper Eyelid; (**B**) Orbital Roof – Extraocular Muscles; (**C**) Orbital Roof – Lower Eyelid; (**D**) Lower Eyelid – Extraocular Muscles

Strong positive correlation were revealed between orbital floor and medial wall (*r* = 0.68; *p* = 0.027); orbital floor and lateral wall (*r* = 0.67; *p* = 0.033); orbital roof and medial wall (*r* = 0.68; *p* = 0.029); lateral wall and medial wall (*r* = 0.75; *p* = 0.013) symmetry parameters (Fig. 11). Among the soft-tissue structures strong negative correlation was found between extraocular muscles and lower eyelid symmetry (*r* = − 0.76; *p* = 0.01). Significant negative correlation was found between orbital floor and upper eyelid (*r* = − 0.72; *p* = 0.019) and positive correlation between orbital roof and extraocular muscles (*r* = 0.67; *p* = 0.033) symmetry parameters (Fig. 12).

## Discussion

Mirroring- and volume-based orbital symmetry assessment remains the most commonly used approach to orbital symmetry analysis [5–7, 13–15]. Interpreting results of the current volume-based orbital symmetry analysis methods may be challenging and confusing in clinical settings. Thus, in cases of orbital trauma, analyzing absolute values of the orbital volume seems insufficient because of the potential shifting of the orbital soft-tissue complex, which volume characteristics may remain unchanged [24–26]. Furthermore, symmetrical enlargement of the orbital fat, extraocular muscles, and lacrimal glands may result in bilateral and barely informative orbital soft-tissue volume increase [5, 7–12, 16]. Differential involvement of the orbital soft-tissue structures in patients with Grave’s disease underlines the importance of their differential symmetry assessment [9–12]. In recent years, interest in surface-based hard- and soft-tissue symmetry evaluation has dramatically increased [1–4, 18, 19]. Interpreting the revealed relative symmetry parameters of the orbital structures, which provides more comprehensible information than analyzing the absolute values of mean and RMS differences between mirrored and registered surfaces, is a reasonable step in developing the surface-based symmetry analysis techniques reported previously [1–4, 18–20], with potential clinical and research applications.

Detecting clinically significant asymmetry zones in all the orbital hard- and soft-tissue structures in all observations supports the concept of preexisting natural facial asymmetry [1–4].

The strong negative correlation between global and local symmetry parameters and RMS distance values makes sense, confirming that the more the distance between the registered true and mirrored model surfaces, the less the global symmetry of the model is (Fig. 10). These findings demonstrate the reliability of the suggested novel approach based on the relative symmetry parameters assessment.

The correlation between the different orbital wall symmetry was higher than between the soft-tissue structures (Fig. 11), reflecting strong structural anatomical connections between the orbital walls. The dynamic and independent behavior of the orbital soft-tissue components can additionally support these findings. Further studies are necessary for appropriate interpretation of the revealed inter-group correlations. That would be a reasonable step towards better understanding the relationship between hard- and soft-tissue symmetry, and acceptance or rejection of the hypothesis on soft-tissue compensation for bone asymmetry [1, 2].

The main limitation of this study is the small sample size, highlighting the need for further research with a larger cohort to fully validate this hypothesis. Nevertheless, it provides valuable insights as a pilot study proving the concept of the separate analysis of the orbital structures and demonstrating strong correlations between certain hard- and soft-tissue structures symmetry parameters. These findings suggest potential compensation of intra- and periorbital soft tissue for bone structure asymmetry, emphasizing the importance of further investigation. Furthermore, anatomy-based standards for orbital soft-tissue structures’ automated segmentation are still lacking. Currently used semi-automated threshold-based techniques still require manual delineation and refinement, increasing time- and labor consumption.

Orbital symmetrical differences among populations with different characteristics such as BMI, and clinical validation through implementation in oral and maxillofacial surgery could offer further valuable insights. Additionally, applying the atlas-based anatomical mapping of the orbit could be a valuable tool for virtual surgical planning in orbital reconstructive surgery. Implementation of the surface-based symmetry analysis may significantly enhance the objectiveness of the orbital reconstruction results postoperative evaluation. Early detection and surface-based analysis of the differential orbital soft-tissue structures involvement in TAO patients may significantly impact treatment results. Moreover, the suggested symmetry analysis approach could be a valuable tool for intra-subject treatment response analysis in this group of patients. Combining MRI-based segmentation of the orbital soft-tissue structures and the proposed symmetry assessment approach could be another future direction for this research.

## Conclusion

Current validation of the novel approach to orbital hard- and soft-tissue symmetry analysis based on qualitative and quantitative interpretation demonstrates its high reliability and potential clinical applicability for orbital hard- and soft-tissue structures evaluation.

Aside from the accurate RMS difference calculation, the global and local symmetry analysis presented in this pilot study offers valuable, reliable, and comprehensible information on the contribution of the particular orbital hard- and soft-tissue structures to facial symmetry.

## Data Availability

No datasets were generated or analysed during the current study.
